# Quantifying years of life lost in Australia: a multiple cause of death analysis

**DOI:** 10.1093/ije/dyae177

**Published:** 2025-01-26

**Authors:** Grace Joshy, Karen Bishop, Hang Li, Lauren Moran, Michelle Gourley, Jennifer Welsh, Rosemary Korda, Emily Banks, Tim Adair, Chalapati Rao

**Affiliations:** National Centre for Epidemiology and Population Health, Australian National University, Canberra, Australia; National Centre for Epidemiology and Population Health, Australian National University, Canberra, Australia; Nossal Institute for Global Health, Melbourne School of Population and Global Health, University of Melbourne, Melbourne, Australia; Australian Bureau of Statistics, Canberra, Australia; Australian Institute of Health and Welfare, Canberra, Australia; National Centre for Epidemiology and Population Health, Australian National University, Canberra, Australia; National Centre for Epidemiology and Population Health, Australian National University, Canberra, Australia; National Centre for Epidemiology and Population Health, Australian National University, Canberra, Australia; Nossal Institute for Global Health, Melbourne School of Population and Global Health, University of Melbourne, Melbourne, Australia; National Centre for Epidemiology and Population Health, Australian National University, Canberra, Australia

**Keywords:** Mortality, years of life lost, cause of death, fatal burden, Australia

## Abstract

**Background:**

Deaths in Australia and other high-income countries increasingly involve multiple conditions. However, key burden of disease measures typically only use the underlying cause of death (UC). We quantified sex and cause-specific years of life lost (YLL) based on UC compared with a method integrating multiple causes of death.

**Methods:**

Causes of death for all deaths in Australia (2015–17), mapped to 136 groups based on International Classification of Diseases 10th revision (ICD-10), were ascribed using (1) the UC only and (2) a multiple cause weighting (WT) strategy. Applying the Global Burden of Disease 2010 life table, YLL_UC_ and YLL_WT_ rates were calculated for each sex and cause of death and compared using relative and absolute measures.

**Results:**

All-cause YLL rates were 113.4/1000 for males and 79.9/1000 for females. Cancers, cardiovascular diseases, external causes, respiratory diseases and nervous system diseases were the five biggest contributors to YLL for each method. For the top 20 causes combined, YLL_WT_ rates were 10% lower for males (YLL_WT_ = 74.93/1000 vs YLL_UC_ = 67.38/1000) and 7% lower for females (YLL_WT_ = 51.34/1000; YLL_UC_ = 47.90/1000); YLL_WT_ rates were lower for ischaemic heart disease and all cancers, but higher for diabetes and dementia, and for chronic obstructive pulmonary disease in males. With multiple cause weighting, renal failure emerged among the top 20 causes of YLL, as did atrial fibrillation and hypertension among females. YLL_WT_ rates for substance abuse, mood disorders, hypertension and schizophrenia were relatively high compared with YLL_UC_.

**Conclusion:**

The YLL_WT_ metric highlights epidemiologically important conditions that are less often selected as the UC.

Key Messages

- This work extends the application of multiple causes of death analysis methods to estimation of fatal burden of disease, quantifies sex and cause-specific years of life list (YLL) using a multiple cause weighting strategy, compares rates with those based only on the underlying cause and discusses implications of integrating multiple causes of death into estimation of fatal burden.
- The weighting strategy applied in this study highlights the importance of several conditions that would remain under-represented in fatal burden of disease metrics if only conventional unweighted measures were applied.
- To our knowledge, no previous study has applied a multiple cause of death approach to population-level estimation of fatal burden, internationally.

## Introduction

Globally, the proportion of deaths attributable to non-communicable diseases (NCDs) increased from almost 61% in 2000 to almost 74% in 2019, accompanying reductions in the proportion due to communicable disease.^1^ As more individuals avoid or survive communicable diseases and other contributors to early deaths, such as maternal and reproductive health issues, they are surviving to older ages where NCDs become the principal health risks. In Australia, as in other high-income countries, this trend has contributed to increasing prevalence of chronic disease multimorbidity, increasing number of years lived in ill health and deaths involving multiple conditions.^2–4^ Presently, only a minority of deaths are due to a single cause; in 2021, only 20% of natural deaths in Australia were due to a single cause,^4^ and deaths from coronary heart disease and diabetes had 3.8 and 5.5 causes recorded on average, respectively.

Multiple cause of death data, which comprise all diseases and conditions contributing to death, are increasingly being used to better understand mortality patterns of multimorbid conditions,^5^ including to estimate mortality rates by ‘any mention’ of cause of death, pairwise combinations or clusters of causes and application of weights to multiple causes of death. However, other key measures of burden of disease, such as years of life lost (YLL), are most commonly based on a single underlying cause of death (UC). Estimates of YLL, that measure fatal burden of disease, are widely used with years lived with disability (YLD), that measure non-fatal burden, to calculate disability-adjusted life years, including by the Global Burden of Disease (GBD) Study^6^ and the Australian Burden of Disease Study^3^ to inform policy and planning. In the context of increasing prevalence of multimorbid chronic diseases, YLD estimates account for comorbid conditions but YLL estimates have only ever been measured using a single UC. Hence existing measures of fatal burden do not represent the contribution of multimorbid conditions, which limits their utility as evidence for health policy. The implication of incorporating multiple causes of death into YLL calculations is not known.

To address this knowledge gap, this study aims to quantify population-level YLL in Australia, comparing sex and cause-specific YLL based on the UC (YLL_UC_) with that incorporating multiple cause weighting strategy (YLL_WT_). Findings are expected to provide new insights into contributions of comorbid conditions to mortality in Australia and is of methodological relevance to other countries collecting data on multiple causes of death.

## Methods

This study used causes of death unit-record files for all deaths in Australia during 2015–17, including International Classification of Diseases 10th Revision (ICD-10) coded causes of death (Supplementary Methods, available as Supplementary data at *IJE* online). All causes were mapped to 136 mutually exclusive and exhaustive groups of health conditions (Supplementary Table S2, available as Supplementary data at *IJE* online), as outlined previously.^7^ Death records with missing age at death (<0.01%) and deaths with an ill-defined UC (as defined in Supplementary Table S2, available as Supplementary data at *IJE* online, <2%) were excluded.

Two different methods of ascribing cause of death were used: the UC only and a multiple cause weighting (WT) strategy (Supplementary Methods, available as Supplementary data at *IJE* online), which ascribed 50% weight to the UC and apportioned the remaining 50% equally across the contributing causes reported in Part II of the death certificate, so that the total sum of weights within each death is 100%.^7^^,^^8^ As illustrated in previous work,^7^ ascribing a weight of 50% to the UC maintains the importance of the UC while considering the overall involvement of contributing causes. As the focus of this study is comparison between UC and WT methods, and methods for redistribution of contributing causes are yet to be developed, we chose not to redistribute ill-defined causes of death as done in the Australian Burden of Disease Study or GBD Study analyses.^9^^,^^10^

### Statistical methods

To calculate years of life lost (YLL), this study applied the standard life table by single year of age used in the 2010 GBD Study^11^ which has a life expectancy at birth of 86.02 years for males and females. The normative standard life table used in the GBD Study was developed by identifying the lowest observed death rate for any age group in countries of more than 5 million in population. Although the more recent 2019 GBD Study^6^ uses an updated abridged life table, we used the life table by single year from the 2010 GBD Study, thus enabling greater precision in YLL estimates. Further, the 2010 GBD Study life table is used by the Australian Institute of Health and Welfare in the Australian Burden of Disease Study, which was used for comparative purposes.^6^^,^^9^

YLL were calculated separately for the two methods of ascribing cause of death. First, the number of age- and cause-specific deaths were calculated using the UC and the weighted multiple causes.^7^ For each sex s and cause of death i, YLL were calculated as:

$${\mathrm{YLL}}_{si}=\sum_{a}D_{\mathrm{sia}}W_{a}$$

where $D_{a}$ is the number of deaths (either UC or WT) at each age a in single years and $W_{a}$ is the YLL weight for deaths at age *a* according to the reference life table (as distinct from the multiple cause weighting WT) (Supplementary Table S1, available as Supplementary data at *IJE* online).^9^ Crude YLL rates (expressed as YLL per 1000 population) based on UC and WT methods were summarized by broad cause groups to provide a higher-level view, and by 20 leading causes of YLL for a more granular assessment. YLL rates were compared using the relative and absolute differences between the UC and WT methods. Crude rates were used for primary comparisons, but age-standardized rates were calculated in Supplementary Analyses, available as Supplementary data at *IJE* online; rates were directly standardized to the 2011 Australian Estimated Resident Population in 5-year age groups (0–4 to 95+). All analysis was undertaken using SAS software version 9.4.

## Results

A total of 469 658 deaths registered over 2015–17 were used in the study—242 039 among males and 227 619 among females. The median age at death was 78 years for males (interquartile range 67–86 years) and 80 years for females (interquartile range 73–91 years). Considering all-cause mortality, total number of YLL were 7 010 874; 4 087 789 for males and 2 923 085 for females. All-cause YLL rates, which do not consider causes of death involved, were 41% higher in males than that in females (113.4 vs 79.9 per 1000).

Considering broad cause groups, neoplasms (cancers), cardiovascular diseases, external causes, respiratory diseases and nervous system diseases were the five biggest contributors to YLL according to both methods—contributing at least 76% of the total from all causes by UC and 82% of that by WT (Tables 1 and 2). While ranking of YLL rates by broad cause groups was somewhat similar by UC and WT methods, there were notable differences between rates of YLL by the two methods. In males, YLL rates by WT were at least one death per 1000 higher than that by UC for deaths from mental and behavioural disorders (by 4.8/1000, 196%), endocrine disorders (by 1.6/1000, 36%) and genitourinary diseases (by 1.1/1000, 75%), while they were at least one death per 1000 lower for deaths from external causes (by 5.0/1000, 26%) and neoplasms (by 4.3/1000, 12%) (Table 1); differences for other condition groups (including cardiovascular diseases, respiratory diseases and nervous system diseases) ranged from −0.02 to +0.3/1000. Generally, the patterns of change in rates were similar in females, but the differences were lower in magnitude for females compared to males (Table 2). Although large relative changes were observed for deaths from hearing and vision conditions (433% for males, 586% for females), the corresponding contribution to YLL in absolute terms was very low (YLL_UC_ = 0.1/1000).

**Table 1.** Years of life lost (YLL) from broad cause groups for males by underlying cause of death (UC) and multiple cause weighting strategy (WT), Australia, 2015–2017

Broad cause groups	Deaths_UC_	YLL_UC_			YLL_WT_			YLL_WT_ − YLL_UC_	
		Rank	Crude rate	% Contribution	Rank	Crude rate	% Contribution	Absolute difference	% Change from YLL_UC_
Neoplasms	78 461	1	36.83	32.5%	1	32.57	28.7%	−4.26	−12%
Cardiovascular diseases	63 472	2	23.72	20.9%	2	24.04	21.2%	0.32	+1%
External causes	20 423	3	19.59	17.3%	3	14.56	12.8%	−5.03	−26%
Respiratory diseases	22 840	4	7.87	6.9%	4	7.96	7.0%	0.09	+1%
Nervous system diseases	22 140	5	7.12	6.3%	5	7.46	6.6%	0.34	+5%
Endocrine disorders	10 429	6	4.62	4.1%	7	6.26	5.5%	1.64	+36%
Digestive diseases	6843	7	2.88	2.5%	8	3.00	2.6%	0.12	+4%
Mental & behavioural disorders	3461	8	2.42	2.1%	6	7.18	6.3%	4.76	+196%
Perinatal conditions (incl SIDS)	996	9	2.37	2.1%	10	2.38	2.1%	0.00	+0.2%
Infectious diseases	4234	10	1.99	1.8%	11	1.98	1.7%	−0.02	−1%
Congenital conditions	979	11	1.50	1.3%	12	1.50	1.3%	0.00	−0.2%
Genitourinary diseases	4902	12	1.42	1.2%	9	2.48	2.2%	1.06	+75%
Musculoskeletal conditions	1422	13	0.52	0.5%	13	0.86	0.8%	0.34	+64%
Blood diseases	736	14	0.35	0.3%	14	0.49	0.4%	0.14	+39%
Skin diseases	676	15	0.22	0.2%	16	0.25	0.2%	0.03	+14%
Hearing & vision diseases	25	16	0.01	0.01%	17	0.06	0.05%	0.05	+433%
Injuries—consequences	–	–	–	–	15	0.43	0.4%	–	–
All-cause	242 039		113.44	100%		113.44	100%		

YLL rates are YLL per 1000. There were 4009 deaths in males with an ill-defined underlying cause of death; those records have been excluded.

% Contribution represents the proportion of cause group specific YLL out of total YLL.

International Classification of Diseases 10th revision (ICD-10) codes used to group causes of death are presented in Supplementary Table S2, available as Supplementary data at *IJE* online.

Age-standardized rates are presented in Supplementary Table S3, available as Supplementary data at *IJE* online.

**Table 2.** Years of life lost (YLL) from broad cause groups for females by underlying cause of death (UC) and multiple cause weighting strategy (WT), Australia, 2015–2017

Broad cause groups	Deaths_UC_	YLL_UC_			YLL_WT_			YLL_WT_ − YLL_UC_	
		Rank	Crude rate	% Contribution	Rank	Crude rate	% Contribution	Absolute difference	% Change from YLL_UC_
Neoplasms	60 855	1	28.51	35.7%	1	25.69	32.1%	−2.82	−10%
Cardiovascular diseases	65 639	2	15.94	19.9%	2	16.07	20.1%	0.13	+1%
External causes	11 694	3	7.49	9.4%	5	5.67	7.1%	−1.82	−24%
Nervous system diseases	31 589	4	7.42	9.3%	3	7.65	9.6%	0.23	+3%
Respiratory diseases	22 347	5	6.54	8.2%	4	6.39	8.0%	−0.15	−2%
Endocrine disorders	9847	6	3.33	4.2%	6	4.48	5.6%	1.15	+35%
Digestive diseases	7902	7	2.41	3.0%	8	2.31	2.9%	−0.10	−4%
Perinatal conditions (incl SIDS)	779	8	1.82	2.3%	10	1.84	2.3%	0.01	+1%
Infectious diseases	4088	9	1.43	1.8%	12	1.27	1.6%	−0.16	−11%
Genitourinary diseases	5813	10	1.35	1.7%	9	2.07	2.6%	0.72	+54%
Congenital conditions	835	11	1.23	1.5%	13	1.21	1.5%	−0.02	−2%
Mental & behavioural disorders	1745	12	1.05	1.3%	7	2.93	3.7%	1.88	+180%
Musculoskeletal conditions	2673	13	0.81	1.0%	11	1.31	1.6%	0.50	+62%
Blood diseases	817	14	0.32	0.4%	14	0.44	0.5%	0.12	+39%
Skin diseases	942	15	0.24	0.3%	15	0.26	0.3%	0.03	+11%
Maternal conditions	25	16	0.04	0.05%	18	0.05	0.1%	0.01	+22%
Hearing & vision diseases	29	17	0.01	0.01%	17	0.06	0.1%	0.05	+586%
Injuries—consequences	–	–	–	–	16	0.25	0.3%	–	–
All-cause	227 619		79.92	100%		79.92	100%		

YLL rates are YLL per 1000. There were 4787 deaths in females with an ill-defined underlying cause of death; those records have been excluded.

% Contribution represents the proportion of cause group specific YLL out of total YLL.

International Classification of Diseases 10th revision (ICD-10) codes used to group causes of death are presented in Supplementary Table S2, available as Supplementary data at *IJE* online.

Age-standardized rates are presented in Supplementary Table S3, available as Supplementary data at *IJE* online.

Tables 3 and 4 show ranking and magnitude of leading causes of YLL by WT vs UC methods. Preventable chronic conditions such as cardiovascular conditions, cancers, hypertension, diabetes, chronic obstructive pulmonary disease (COPD), dementia and renal failure, as well as alcohol-induced diseases and external causes (e.g. suicides, accidental poisoning-drugs) were highly represented. The top 20 causes combined contributed at least 60% of the total YLL; rates by WT were 10% lower for males (YLL_WT_ = 74.93/1000; YLL_UC_ = 67.38/1000) and 7% lower for females (YLL_WT_ = 51.34/1000; YLL_UC_ = 47.90/1000). The leading cause of YLL using both methods was ischaemic heart disease (YLL_UC_ 12.9/1000 among males and 6.0/1000 among females), with rates being lower under WT [by 1.5/1000 (11%) among males and by 0.9/1000 (15%) among females]. Other causes of YLL ranked among the top five under both methods included suicide (among males), lung cancer, cerebrovascular disease, breast cancer (among females) and dementia & Alzheimer’s disease (among females).

**Table 3.** Top 20 causes of years of life lost (YLL) for males by underlying cause of death (UC) and multiple cause weighting strategy (WT), Australia, 2015–2017

Cause	Deaths_UC_	YLL_UC_			YLL_WT_			YLL_WT_ − YLL_UC_	
		Rank	Crude rate	% Contribution	Rank	Crude rate	% Contribution	Absolute difference	% Change from YLL_UC_
Ischaemic heart disease	32 634	1	12.87	11.3%	1	11.4	10.0%	−1.47	−11%
Suicide	6850	2	8.04	7.1%	3	5.23	4.6%	−2.81	−35%
Lung cancer	14 926	3	7.14	6.3%	2	5.94	5.2%	−1.2	−17%
Cerebrovascular disease	12 910	4	4.21	3.7%	7	3.65	3.2%	−0.56	−13%
Colorectal cancer	8782	5	4.20	3.7%	6	3.75	3.3%	−0.45	−11%
COPD	11 764	6	4.13	3.6%	4	4.31	3.8%	0.17	+4%
Dementia & Alzheimer’s disease	14 711	7	3.44	3.0%	8	3.56	3.1%	0.12	+4%
Diabetes	7651	8	3.15	2.8%	5	4.11	3.6%	0.96	+31%
Prostate cancer	9722	9	3.12	2.8%	9	2.91	2.6%	−0.21	−7%
Accidental poisoning-drugs^a^	2226	10	2.77	2.4%	20	1.70	1.5%	−1.07	−39%
Other blood cancers	6171	11	2.66	2.3%	11	2.38	2.1%	−0.28	−11%
Residual-external causes	3070	12	2.43	2.1%	14	2.01	1.8%	−0.42	−17%
Perinatal conditions (incl SIDS)^a^	996	13	2.37	2.1%	11	2.38	2.1%	0	+0.20%
Pancreatic cancer	4501	14	2.25	2.0%	15	1.95	1.7%	−0.3	−13%
Alcohol induced diseases^a^	2796	15	2.15	1.9%	10	2.87	2.5%	0.72	33%
Other heart diseases	3844	16	2.07	1.8%	13	2.07	1.8%	0	+0.10%
Liver cancer	3695	16	2.07	1.8%	18	1.76	1.5%	−0.31	−15%
RTI: motor vehicle occupants^a^	1676	18	2.04	1.8%	17	1.81	1.6%	−0.22	−11%
Cancer unknown primary	4319	19	1.93	1.7%	*(*22*)*	*(1.64)*	*(1.4%)*	−0.29	−15%
Brain cancer^a^	2582	20	1.89	1.7%	19	1.73	1.5%	−0.16	−8%
Renal failure	3193	*(*34*)*	*(0.90)*	*(0.8%)*	*16*	1.85	1.6%	0.95	+105%
Top 20 causes combined			74.93	66.1%		67.38	59.4%	−7.55	−10%
All causes			113.44			113.44			

YLL rates are YLL per 1000. % Contribution represents the proportion of cause-specific YLL out of total YLL. International Classification of Diseases 10th revision (ICD-10) codes corresponding to causes of death are presented in Supplementary Table S2, available as Supplementary data at *IJE* online. Ranks and rates in italics are not among the top 20 but are presented here for completeness. Lowest rank was assigned in case of tied rates.

a Not among the top 20 underlying causes of death. Ranks, crude rates and age-standardized rates for all-cause groups are presented in Supplementary Table S4, available as Supplementary data at *IJE* online.

**Table 4.** Top 20 causes of years of life lost (YLL) for females by underlying cause of death (UC) and multiple cause weighting strategy (WT), Australia, 2015–2017

Cause	Deaths_UC_	YLL_UC_			YLL_WT_			YLL_WT_ − YLL_UC_	
		Rank	Crude rate	% Contribution	Rank	Crude rate	% Contribution	Absolute difference	% Change from YLL_UC_
Ischaemic heart disease	25 051	1	5.95	7.4%	1	5.07	6.3%	−0.88	−15%
Lung cancer	10 216	2	4.96	6.2%	4	4.21	5.3%	−0.75	−15%
Breast cancer	8815	3	4.89	6.1%	3	4.62	5.8%	−0.27	−5%
Dementia & Alzheimer’s disease	26 142	4	4.79	6.0%	2	4.90	6.1%	0.11	+2%
Cerebrovascular disease	18 611	5	4.46	5.6%	5	3.76	4.7%	−0.71	−16%
COPD	10 174	6	3.37	4.2%	6	3.31	4.1%	−0.06	−2%
Colorectal cancer	7557	7	3.16	4.0%	7	2.85	3.6%	−0.31	−10%
Suicide^a^	2282	8	2.64	3.3%	10	1.63	2.0%	−1.00	−38%
Diabetes	6659	9	2.14	2.7%	8	2.70	3.4%	0.57	+27%
Perinatal conditions (incl SIDS)^a^	779	10	1.82	2.3%	9	1.84	2.3%	0.01	+1%
Pancreatic cancer	4170	11	1.78	2.2%	12	1.56	1.9%	−0.22	−12%
Other blood cancers	4378	12	1.76	2.2%	11	1.57	2.0%	−0.19	−11%
Ovarian cancer	2858	13	1.45	1.8%	15	1.31	1.6%	−0.14	−10%
Cancer unknown primary	3808	14	1.42	1.8%	16	1.21	1.5%	−0.20	−14%
Congenital conditions^a^	835	15	1.23	1.5%	16	1.21	1.5%	−0.02	−2%
Brain cancer^a^	1663	16	1.16	1.5%	20	1.08	1.3%	−0.08	−7%
Residual-malignant neoplasms*	2213	17	1.14	1.4%	*(*21*)*	*(1.02)*	*(1.3%)*	−0.12	−10%
Other heart diseases	2962	17	1.14	1.4%	19	1.11	1.4%	−0.02	−2%
Pneumonia	4795	19	1.07	1.3%	*22*	0.98	1.2%	−0.09	−8%
Accidental poisoning-drugs^a^	947	20	1.06	1.3%	*(*35*)*	*(0.66)*	*(0.8%)*	−0.40	−38%
Renal failure	3515	*(*26*)*	*(0.80)*	*(1.0%)*	13	1.47	1.8%	0.67	+84%
Atrial fibrillation	4080	*(*28*)*	*(0.75)*	*(0.9%)*	18	1.12	1.4%	0.38	+51%
Hypertension^a^	1150	*(*66*)*	*(0.22)*	*(0.3%)*	14	1.36	1.7%	1.14	+516%
Top 20 causes combined			51.34	64.2%		47.90	59.9%	−3.44	−7%
All causes			79.92			79.92			

YLL rates are YLL per 1000. % Contribution represents the proportion of cause-specific YLL out of total YLL. International Classification of Diseases 10th revision (ICD-10) codes corresponding to causes of death are presented in Supplementary Table S2, available as Supplementary data at *IJE* online. Ranks and rates in italics are not among the top 20 but are presented here for completeness. Lowest rank was assigned in case of tied rates.

a Not among the top 20 underlying causes of death. Ranks, crude rates and age-standardized rates for all-cause groups are presented in Supplementary Table S4, available as Supplementary data at *IJE* online.

YLL_WT_ rates were substantially lower than YLL_UC_ for all leading external causes of YLL, most notably deaths from suicide (by 35% for males and 38% for females) and accidental poisoning by drugs (by 39% for males and 38% for females), as well as for cerebrovascular disease (by 13% for males and 16% for females). Similarly, for the leading cancer causes, YLL_WT_ rates were lower than YLL_UC_ in males and females for lung (by 17% and 15% respectively), pancreatic (by 13% and 12%) and colorectal (by 11% and 10%). Among males, rates by YLL_WT_ were lower than YLL_UC_ for prostate cancer (by 7%) and liver cancer (by 15%). Among females, rates by YLL_WT_ were lower than YLL_UC_ for breast cancer (by 5%) and ovarian cancer (10% lower). Among the top 20 causes of YLL, YLL_WT_ rates were higher than YLL_UC_ for deaths from diabetes (by 31% for males and 27% for females), dementia (by 4% and 2%), renal failure—also known as chronic kidney disease—(by 105% and 84%) and COPD among males (4%); the corresponding YLL_UC_ rates ranged between 2/1000 and 5/1000 (Tables 3 and 4). Renal failure was not among the top 20 causes of YLL based on UC, but its rate was substantially higher under the WT method (YLL_UC_ = 0.9/1000 vs YLL_WT_ = 1.85/1000, a 105% increase for males; YLL_UC_ = 0.8/1000 vs YLL_WT_ = 1.5, an 84% increase for females). Under WT, atrial fibrillation and hypertension also emerged among the top 20 causes of YLL for females.

**Figure 1.**
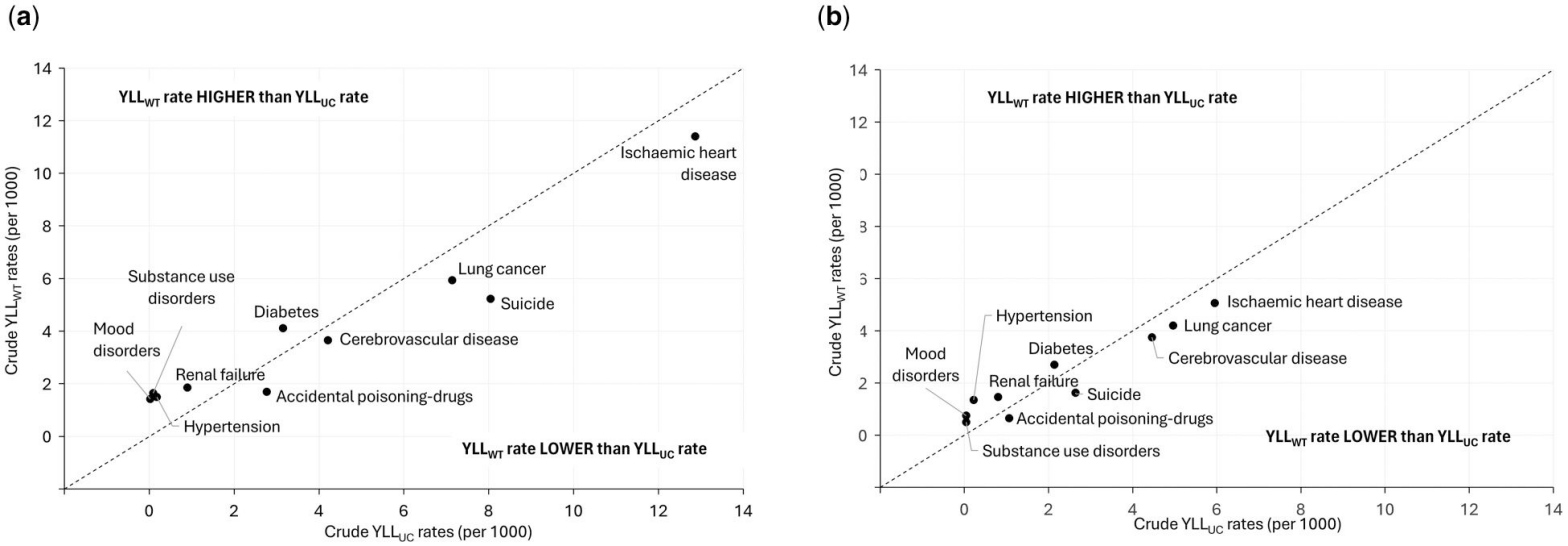
Top 10 causes with largest absolute increase or absolute decrease from years of life lost based on underlying cause of death (YLL_UC_) to years of life lost based on multiple cause weighting (YLL_WT_) by sex: (a) Males, (b) Females. Figure represents the ranking of cause groups by absolute difference (years of life lost based on multiple cause weighting (YLL_WT_) − years of life lost based on underlying cause of death (YLL_UC_)); five cause groups with the largest absolute increase and five with the largest absolute decrease are plotted. Causes above the diagonal dotted line (e.g. diabetes) make a larger contribution to Australia’s total years of life lost (YLLs) when rates are measured using multiple cause of weighting compared with using a single underlying cause. Conversely, causes below the diagonal dotted line (e.g. ischaemic heart disease) make a smaller contribution to Australia’s total YLLs when rates are measured using multiple cause of weighting compared with using a single underlying cause. Among causes not captured in the top 20 causes of YLL (ranking by YLL rates, Tables 1 and 2), large absolute increase in YLL_WT_ compared to YLL_UC_ were observed for mood disorders and substance use disorders

**Figure 2.**
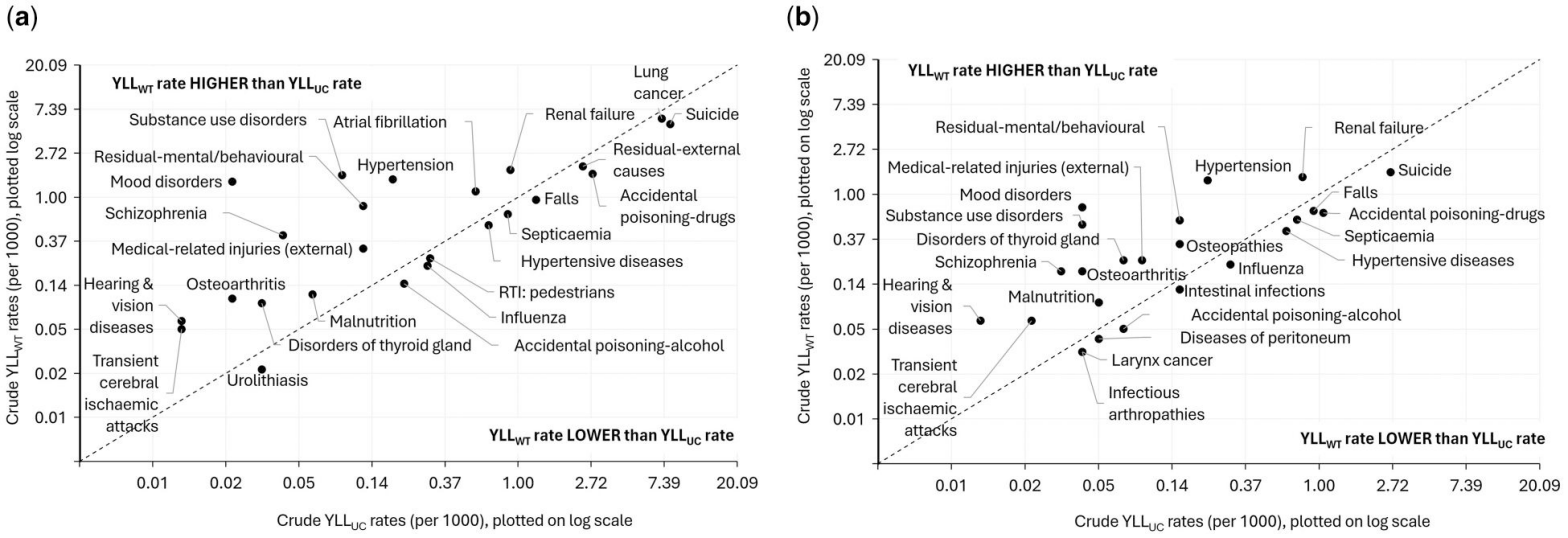
Top 10 causes with largest relative increase and top 10 causes with largest relative decrease from years of life lost based on underlying cause of death (YLL_UC_) to years of life lost based on multiple cause weighting (YLL_WT_) by sex: (a) Males, (b) Females. Figure is based on ranking of cause groups by relative difference (% change from years of life lost based on underlying cause of death (YLL_UC_)); 10 cause groups with the largest relative increase and 10 with the largest relative decrease are plotted. Large relative increase in years of life lost based on multiple cause weighting strategy (YLL_WT_) compared to YLL_UC_ was observed for deaths from several conditions, including atrial fibrillation, hypertension, substance abuse, mood disorders, schizophrenia, osteoarthritis, metabolic disorders and transient cerebral ischaemic attack. Causes with large relative decrease in YLL_WT_ compared to YLL_UC_ include suicide, accidental poisoning by drugs and lung cancer (in males), as well as for septicaemia, falls and hypertensive diseases. Diseases of peritoneum and intestinal infections were ties for ranking by relative decrease among females, and both have been plotted. As the top 10 causes with the largest relative increase included external causes, non-specific causes and conditions unlikely to result in death (medical-related injuries (external), residual-mental/behavioural and hearing & vision diseases respectively), causes with the next largest relative increase (renal failure, atrial fibrillation and malnutrition among males; osteopathies, renal failure and malnutrition among females) have also been plotted

Among causes not captured in the top 20 causes of YLL, large absolute increase in YLL_WT_ compared to YLL_UC_ was observed for mood disorders and substance use disorders (Figure 1a and b), and high relative increase in YLL_WT_ compared to YLL_UC_ was observed for deaths from several conditions, including atrial fibrillation, hypertension, substance abuse, mood disorders, Schizophrenia, osteoarthritis, metabolic disorders and transient cerebral ischaemic attack (Figure 2a and b).

The observed patterns in differences between the two methods were similar regardless of whether crude or age-standardized YLL rates were used (Supplementary Tables S3 and S4, available as Supplementary data at *IJE* online).

## Discussion

This study provides comprehensive population-level estimates of YLL in Australia, and new insights into the contribution of multiple conditions to fatal burden in Australia that have not been shown by previous analyses of YLL. To our knowledge, no previous study has applied a multiple cause of death approach to population-level estimation of fatal burden, internationally. Neoplasms, cardiovascular diseases, external causes, respiratory diseases and nervous system diseases consistently ranked among the five biggest contributors to YLL by UC and WT, but there were notable differences between rates by both methods. Considering the top 20 causes of YLL, rates dropped with multiple cause weighting for ischaemic heart disease, suicide, accidental poisoning by drugs, and cancers in general, while they were higher for deaths from diabetes, dementia, renal failure and COPD among males. With multiple cause weighting, renal failure emerged among the top 20 causes of YLL among males while cancer of unknown primary dropped out; renal failure, atrial fibrillation and hypertension emerged among the top 20 causes of YLL among females while residual malignant neoplasms, pneumonia and accidental poisoning by drugs dropped out. YLL rates were also relatively higher with multiple cause weighting for metabolic disorders, atrial fibrillation, obesity, substance abuse, mood disorders and osteoarthritis.

The most prominent use of YLL in analysis of Australian mortality to date have been by the GBD Study and Australian Burden of Disease Study, both of which measure YLL using only the UC.^6^^,^^9^^,^^12^ Consistent with findings from Australian Burden of Disease Study and GBD Study, leading causes of YLL_UC_ include cancers, cardiovascular disease, suicide, COPD, dementia & Alzheimer’s disease and diabetes, while conditions such as renal failure, hypertension and atrial fibrillation were not represented in leading causes by YLL_UC_. It should be noted that the rates from our study are not directly comparable to those from the GBD Study or the Australian Burden of Disease Study; unlike our study, GBD Study and Australian Burden of Disease Study redistribute ill-defined or unspecified causes of death to other causes, often using algorithms informed by multiple cause of death data.

To our knowledge, this is the first study to measure YLL across all causes of death using a multiple cause approach. While some previous studies have used multiple causes of death in YLL estimation,^13–16^ we could not identify any study on population-level quantification of YLL, considering the UC as well as other contributing causes that were not part of the main morbid process. Unlike estimates of non-fatal burden (YLD), fatal burden estimates (YLL) do not account for comorbid conditions. Multiple cause weighting for YLL allows comorbid conditions to be accounted for in the estimation of fatal burden, reflecting the reality that multiple causes of death can contribute to a person’s premature death, improving estimates of health care costs, productivity burden and interventions.

Cause-specific YLLs by multiple cause weighting reveals the epidemiological importance of hypertension, atrial fibrillation, renal failure and osteoarthritis—conditions that are less often selected as the UC of death. Number of deaths and age at death are key factors that drive YLL. The number of deaths from chronic conditions form a large proportion of total deaths. It is likely that conditions such as obesity, hypertension and renal failure frequently present as contributing causes for a large proportion of deaths, including for deaths from chronic conditions at relatively younger ages.^17^ The multiple cause weighting strategy for YLL proposed in our study can hence be used to monitor trends in premature mortality from such multimorbid chronic diseases, that would otherwise be masked by analysing the UC of death without accounting for the age at death.

The influence of age at death can also be seen for mental health conditions—primarily substance abuse and mood disorders—when listed as contributing causes (in Part II of the death certificate) with other UCs for deaths at younger ages. The marginal declines induced by the weighting approach for YLL from cancers potentially arise from the higher propensity of cancers to be the UC rather than a non-UC, and deaths from cancers commonly being certified with fewer number of conditions on the death certificate across all ages. Lower rates observed using WT compared to the UC approach for suicides should be interpreted with caution. There are inherent differences in how information in Part II is captured in coroner certified deaths (around 10–12% of deaths per year in Australia), including suicides. As the ABS uses the full coronial brief including pathology, police and toxicology reports to capture information, Part II is much more comprehensive for coroner certified deaths. Multiple cause weighting is likely to be informative for injuries which are more often certified as non-underlying causes. For deaths from other leading causes such as cardiovascular diseases and COPD, which usually occur at older ages, compensatory weight accrual from mentions in Part II across all ages result in only a marginal change in the weighted YLL. The weighting strategy applied in this study highlights the importance of several conditions that would remain under-represented in fatal burden of disease metrics if only conventional unweighted measures were applied, without considerably affecting the magnitude of YLL from most other commonly known leading causes of death.

Strengths of this study include use of national-level unit-record data, evaluation of a broad spectrum of causes and first ever application of multiple cause weighting strategy for YLL estimation, maintaining the importance of the UC and overall involvement of contributing causes. This approach helps overcome inconsistent certification practices regarding reporting of a cause in Part I or Part II of the death certificate. Using a weighting approach to estimate YLL reflects the reality that a combination of health conditions contributes to premature mortality, rather than just a sole UC of death. However, use of information from Part II is limited by differences in death certification practices (e.g. certification by a general practitioner vs a specialist) and the extent of under- and over-reporting. Comprehensive data on multiple causes of death are not being collected in many low-and-middle-income countries^5^; wider implementation of the medical certificate of cause of death and collection of data on multiple causes of death will facilitate comprehensive burden of disease analyses incorporating both the UC and contributory causes. It is important to note key differences in methods when drawing comparisons with previous studies—these include redistribution of ill-defined causes in the GBD Study^18^^,^^19^ and the Australian Burden of Disease Study,^9^ life table used and population for age standardization. Specificity of causes included in the cause list, handling of duplicate mentions of causes, the type of weighting strategy used and causes included in weighting,^7^ as well as changes over time in certification practices as in the case of reporting of dementia in Australia^20^ are also important considerations. While the multiple cause weighting approach would benefit from refinement of methods (e.g. redistribution of non-specific contributing causes and the selection of clinically relevant contributing causes for weighting), the weighting approach as applied in this study presents a comprehensive picture of causes contributing to the fatal burden of disease in Australia.

Combination of weighted YLL with non-fatal burden metrics (e.g. YLD) for each cause would allow understanding of the influence of the outlined analytic strategy on national burden of disease estimates, but comparisons are beyond the scope of this study.

## Conclusion

The multiple cause weighting strategy for YLL highlights the epidemiological importance of conditions that are more likely to be contributing causes of mortality than the UC. Many of the leading contributors to YLL are preventable, and our findings incorporating multiple causes of death into estimation of fatal burden provide a more complete picture of burden disease in Australia and informs identification of targets for prevention, potentially leading to improved prevention strategies.

## Ethics approval

Human Ethics Protocol was approved by the ANU Science and Medical Delegated Ethical Review Committee (Protocol number 2019/022).

## Supplementary Material

dyae177_Supplementary_Data

## Data Availability

This study used death records from each of the State and Territory Registries of Births, Deaths and Marriages and from State and Chief Coroners through the National Coronial Information System, with cause of death information coded by the Australian Bureau of Statistics. The underlying data were provided by the Australian Coordinating Registry (ACR) in the Cause of Death Unit Record File for years 2015 to 2017. Restrictions apply to the availability of these data, which were used under licence for the current study, and so are not publicly available. Data are however available to approved users meeting the eligibility requirements through an application process administered by the ACR.
