# Supplementary material for: Quantifying years of life lost in Australia: a multiple cause of death analysis
Source: Int J Epidemiol. 2025 Jan 26;54(1):dyae177. doi: 10.1093/ije/dyae177 (PMC11769717; doi:10.1093/ije/dyae177)
Supplement: dyae177_Supplementary_Data [file dyae177_supplementary_data.zip › 0d2d9_ije-2024-03-0293-File005.docx]

**Supplementary Material**

Table of Contents

[**Supplementary Methods** 2](#_Toc184739729)

[**Supplementary Table S1. Global Burden of Disease (GDB) 2010 Lifetable** 3](#_Toc184739730)

[**Supplementary Table S2. Cause list used to group causes of death** 4](#_Toc184739731)

[**Supplementary Table S3. Number of deaths, crude years of life lost (YLL) rates and age-standardised YLL rates by underlying cause of death (UC) and multiple cause weighting (WT) - broad cause groups by sex, Australia, 2015-17** 9](#_Toc184739732)

[**Supplementary Table S4. Number of deaths, crude years of life lost (YLL) rates and age-standardised YLL rates by underlying cause of death (UC) and multiple cause weighting (WT) – causes of death by sex, Australia, 2015-17** 10](#_Toc184739733)

# **Supplementary Methods**

This study used causes of death unit record files for all deaths in Australia during 2015–17, including age at death, reference year and the International Classification of Diseases 10^th^ Revision (ICD-10) coded causes of death. The causes of death unit record files were supplied by the Australian Coordinating Registry in February 2020. For each death record, causes of death include an ‘entity axis’ and a ‘record axis.

**Entity axis data** contains information on the chain of events leading to death (i.e. the causal sequence certified in Part 1) and other contributing conditions (i.e. information certified in Part II) as they appear on the Medical Certificate of Cause of Death. For deaths that are referred to the coroner, the Australian Bureau of Statistics codes causes of death from information contained on the National Coronial Information System (NCIS), including police, autopsy, toxicology and coroners’ reports. All relevant details from these reports are used to populate Part II. As a result, risk factors and co-morbid conditions are captured differently for coroner certified deaths. For both doctor certified and coroner certified deaths, Part II is likely to contain other relevant conditions contributing to death, but not related to the disease or condition causing it.

**Record axis data** contains a single underlying cause of death (UC) and non-underlying causes (up to 19 in Australia) generated by application of ICD-10 coding and processing rules to all causes reported on the death certificate.

**Multiple cause weighting (WT) strategy**: We used record axis data for UC. Under WT, the record axis data were used to identify the UC and the entity axis data to identify other relevant conditions which have contributed to the death, but which are not deemed part of the morbid train of events leading to the death (reported in Part II of the death certificate). As the focus of this study is comparison between UC and WT methods, and methods for redistribution of contributing causes are yet to be developed, we chose not to redistribute ill-defined causes of death as done in the Australian Burden of Disease Study or Global Burden of Disease (GBD) Study analyses.(9, 10) Ill-defined causes and duplicate mentions (due to mapping to the same cause group) were not considered in deriving the weights. Under the weighting strategy used in this study, if a cause is reported in Part I (but not as the underlying cause) it would not contribute towards years of life lost (YLL) estimates in neither the weighting strategy not the UC strategy.

**Caveats around cause of death unit record files:** A limitation of the data is that, as recently identified by the Australian Bureau of Statistics, cause of death information from Part II of the death certificate for data from Western Australia were not being captured in the system for deaths from 2016-2020; approximately 6% of deaths used in this study may be impacted.

# **Supplementary Table S1. Global Burden of Disease (GDB) 2010 Lifetable**

| **Age at death (years)** | **Life expectancy** |
| --- | --- |
| 0 | 86.02 |
| 1 | 85.21 |
| 2 | 84.22 |
| 3 | 83.23 |
| 4 | 82.24 |
| 5 | 81.25 |
| 6 | 80.25 |
| 7 | 79.26 |
| 8 | 78.26 |
| 9 | 77.27 |
| 10 | 76.27 |
| 11 | 75.28 |
| 12 | 74.28 |
| 13 | 73.29 |
| 14 | 72.29 |
| 15 | 71.29 |
| 16 | 70.3 |
| 17 | 69.32 |
| 18 | 68.33 |
| 19 | 67.34 |
| 20 | 66.35 |
| 21 | 65.36 |
| 22 | 64.37 |
| 23 | 63.38 |
| 24 | 62.39 |
| 25 | 61.4 |
| 26 | 60.41 |
| 27 | 59.43 |
| 28 | 58.44 |
| 29 | 57.45 |
| 30 | 56.46 |
| 31 | 55.48 |
| 32 | 54.49 |
| 33 | 53.5 |
| 34 | 52.52 |
| 35 | 51.53 |
| 36 | 50.56 |
| 37 | 49.58 |
| 38 | 48.6 |
| 39 | 47.62 |
| 40 | 46.64 |
| 41 | 45.67 |
| 42 | 44.71 |
| 43 | 43.74 |
| 44 | 42.77 |
| 45 | 41.8 |
| 46 | 40.85 |
| 47 | 39.9 |
| 48 | 38.95 |
| 49 | 38 |
| 50 | 37.05 |
| 51 | 36.12 |
| 52 | 35.19 |
| 53 | 34.25 |
| 54 | 33.32 |
| 55 | 32.38 |
| 56 | 31.47 |
| 57 | 30.55 |
| 58 | 29.64 |
| 59 | 28.73 |
| 60 | 27.81 |
| 61 | 26.91 |
| 62 | 26 |
| 63 | 25.1 |
| 64 | 24.2 |
| 65 | 23.29 |
| 66 | 22.42 |
| 67 | 21.55 |
| 68 | 20.68 |
| 69 | 19.8 |
| 70 | 18.93 |
| 71 | 18.1 |
| 72 | 17.28 |
| 73 | 16.45 |
| 74 | 15.62 |
| 75 | 14.8 |
| 76 | 14.04 |
| 77 | 13.27 |
| 78 | 12.51 |
| 79 | 11.75 |
| 80 | 10.99 |
| 81 | 10.32 |
| 82 | 9.65 |
| 83 | 8.98 |
| 84 | 8.31 |
| 85 | 7.64 |
| 86 | 7.12 |
| 87 | 6.61 |
| 88 | 6.09 |
| 89 | 5.57 |
| 90 | 5.05 |
| 91 | 4.7 |
| 92 | 4.35 |
| 93 | 4.00 |
| 94 | 3.66 |
| 95 | 3.31 |
| 96 | 3.09 |
| 97 | 2.88 |
| 98 | 2.66 |
| 99 | 2.44 |
| 100 | 2.23 |
| 101 | 2.11 |
| 102 | 1.99 |
| 103 | 1.87 |
| 104 | 1.75 |
| 105 | 1.63 |

# **Supplementary Table S2. Cause list used to group causes of death**

| **Cause name** | **International Classification of Diseases 10^th^ revision (ICD-10) codes** |  |  |
| --- | --- | --- | --- |
| **Infectious diseases** |  |  |  |
| Intestinal infections | A00–A09 |  |  |
| Tuberculosis | A15–A19, B90 |  |  |
| Septicaemia | A40–A41 |  |  |
| Viral hepatitis | B15–B19, B942 |  |  |
| HIV disease | B20–B24 |  |  |
| Residual - infections | A20–A39, A42–A99, B00–B09, B25–B34, B35–B49, B50–B89, B91–B93, B940–B941, B948–B949, B95–B99 |  |  |
| **Neoplasms** |  |  |  |
| Oral cancers | C00–C14 |  |  |
| Oesophagus cancer | C15 |  |  |
| Stomach cancer | C16 |  |  |
| Colorectal cancer | C18–C21, C260 |  |  |
| Liver cancer | C22 |  |  |
| Gallbladder cancer | C23–C24 |  |  |
| Pancreatic cancer | C25 |  |  |
| Larynx cancer | C32 |  |  |
| Lung cancer | C33–C34 |  |  |
| Malignant melanoma-skin | C43 |  |  |
| Non-melanoma-skin | C44 |  |  |
| Mesothelioma | C45 |  |  |
| Breast cancer | C50 |  |  |
| Cervical cancer | C53 |  |  |
| Uterus cancer | C54–C55 |  |  |
| Ovarian cancer | C56 |  |  |
| Prostate cancer | C61 |  |  |
| Kidney cancer | C64 |  |  |
| Bladder cancer | C67 |  |  |
| Brain cancer | C71 |  |  |
| Thyroid cancer | C73 |  |  |
| Cancer unknown primary | C76, C80, C97 |  |  |
| Cancer secondary site | C77–C79 |  |  |
| Hodgkin lymphoma | C81 |  |  |
| Non-Hodgkin lymphomas | C82–C86 |  |  |
| Other blood cancers | C88, C90–C96, D45–D46, D471, D473–D475 |  |  |
| Residual - benign/in situ/uncertain neoplasms | D00–D44, D470, D472, D477–D479, D48 |  |  |
| Residual - malignant neoplasms | C17, C261, C268, C269, C30–C31, C37–C39, C40–C41, C46–C49, C51–C52, C57–C58, C60, C62–C63, C65–C66, C68–C70, C72, C74–C75 |  |  |
| **Blood diseases** |  |  |  |
| Anaemias | D50–D64 |  |  |
| Residual - blood diseases | D65–D89 |  |  |
| **Endocrine disorders** |  |  |  |
| Disorders of thyroid gland | E00–E07 |  |  |
| Diabetes mellitus | E10–E14 |  |  |
| Malnutrition | E40–E46 |  |  |
| Obesity | E66 |  |  |
| Amyloidosis | E85 |  |  |
| Dehydration disorders | E86–E87 |  |  |
| Metabolic disorders | E70–E84, E88–E90 |  |  |
| Residual - endocrine | E15–E16, E20–E243, E248–E35, E50–E65, E67–E68 |  |  |
| **Cause list (continued)** | | | |
| **Cause name** | **ICD-10 codes** | |  |
| **Mental & behavioural disorders** |  | |  |
| Alcohol induced diseases | E244, F10, G312, G621, G721, I426, K292, K70, K852, K860 | |  |
| Substance use disorders | F11–F19 | |  |
| Schizophrenia | F20–F29 | |  |
| Mood disorders | F30–F39 | |  |
| Residual - mental/behavioural | F04–F09, F40–F99 | |  |
| **Nervous system diseases** |  | |  |
| Inflammatory diseases - CNS | G00–G09 | |  |
| Systemic atrophies - CNS | G10–G14 | |  |
| Parkinson disease | G20 | |  |
| Dementia & Alzheimer's disease | F00–F03, G30, G310, G318 | |  |
| Multiple sclerosis | G35 | |  |
| Epilepsy | G40, G41 | |  |
| Cerebral palsy | G80 | |  |
| Residual - nervous system | G21–G26, G311, G319, G32, G36–G37, G43–G44, G47, G50–G620, G622–G64, G70–G720, G722–G73, G81–G83, G90–G99 (G45–G46 – to TIA, G310, G318 to Dementia) | |  |
| **Hearing and vision diseases** | H00–H95 | |  |
| **Cardiovascular diseases** |  | |  |
| Chronic rheumatic heart diseases | I05–I09 | |  |
| Hypertension | I10 | |  |
| Hypertensive diseases | I11–I15 | |  |
| Ischaemic heart disease | I20–I25 | |  |
| Pulmonary heart diseases | I26–I28 | |  |
| Non-rheumatic valve disorders | I34–I36 | |  |
| Atrial fibrillation | I48 | |  |
| Heart failure (specified) | I500, I501 | |  |
| Other heart diseases | I30–I33, I37–I39, I40–I41, I420–I425, I427–I43, I44–I45, I47, I49, I51, I52 | |  |
| Cerebrovascular disease | I60–I69 | |  |
| Artery diseases | I70–I79 | |  |
| Phlebitis & thrombophlebitis | I80 | |  |
| Transient cerebral ischaemic attack | G45–G46 | |  |
| Residual - cardiovascular | I00–I02, I81–I89, I950–I958, I96–I98 | |  |
| **Respiratory diseases** |  | |  |
| Influenza | J09–J11 | |  |
| Pneumonia | J12–J18 | |  |
| Other ALRI | J20–J22 | |  |
| COPD | J40–J44 | |  |
| Asthma | J45–J46 | |  |
| Bronchiectasis | J47 | |  |
| Pneumonitis | J69 | |  |
| Other interstitial respiratory diseases | J80–J84 | |  |
| Other diseases of pleura | J90–J94 | |  |
| Residual - respiratory | J00–J06, J30–J39, J60–J68, J70, J85–J86, J95, J961–J968, J97–J99 | |  |

| **Cause list (continued)** | | | | |
| --- | --- | --- | --- | --- |
| **Cause name** | **ICD-10 codes** | |  |  |
| **Digestive diseases** |  | |  |  |
| Diseases of oesophagus, stomach & duodenum | K20–K291, K293–K31 | |  |  |
| Other diseases of intestines | K55–K64 | |  |  |
| Diseases of peritoneum | K65–K67 | |  |  |
| Cirrhosis of the liver | K74 | |  |  |
| Other diseases of liver | K71–K73, K75–K77 | |  |  |
| Disorders of gallbladder, biliary tract & pancreas | K80–K851, K853–K859, K861–K87 | |  |  |
| Residual - digestive | K00–K14, K35–K38, K40–K46, K50–K52, K90–K93 | |  |  |
| **Skin diseases** |  | |  |  |
| Infections of skin | L00–L08 | |  |  |
| Residual - skin diseases | L10–L99 | |  |  |
| **Musculoskeletal conditions** |  | |  |  |
| Infectious arthropathies | M00–M03 | |  |  |
| Rheumatoid arthritis | M05–M06 | |  |  |
| Osteoarthritis | M15–M19 | |  |  |
| Systemic connective tissue disorders | M30–M36 | |  |  |
| Osteopathies & chondropathies | M80–M94 | |  |  |
| Residual - musculoskeletal | M07–M14, M20–M25, M40–M79, M95–M99 | |  |  |
| **Genitourinary diseases** |  | |  |  |
| Glomerular diseases | N00–N08 | |  |  |
| Renal tubulo-interstitial diseases | N10–N16 | |  |  |
| Renal failure | N17–N19 | |  |  |
| Urolithiasis | N20–N23 | |  |  |
| Hyperplasia of prostate | N40 | |  |  |
| Residual - genitourinary | N25–N29, N30–N39, N41–N51, N60–N99 | |  |  |
| **Maternal conditions** | O00–O99 | |  |  |
| **Perinatal conditions (including SIDS)** | P00–P284, P286–P96, R95 | |  |  |
| **Congenital conditions** | Q00–Q99 | |  |  |
| **Ill-defined causes** | I46, I509, I959, I99, J960, J969, P285, R00–R99 (excl R95) | |  |  |
| **Injuries** | S00–T99 | |  |  |
| Traumatic brain injury | S020, S021, S027, S028, S029, S06, T902, T905 | |  |  |
| Spinal cord injury | S140, S141, S147, S240, S241, S247, S340, S341, S347, T060, T061, T093, T903, T913 | |  |  |
| Internal & crush injuries | S07, S17, S18, S224, S225, S25, S26, S27, S28, S297, S35, S36, S37, S380, S381, S396, S397, S47, S57, S67, S77, S87, S97, T04, T065, T147, T914, T915 | |  |  |
| Poisoning - other substances | T36–T39, T407–T409, T41–T50, T52–T65, T940, T941, T96, T97 | |  |  |
| Poisoning - alcohol | T51 | |  |  |
| Poisoning - opioid | T400–T406 | |  |  |
| Hip fracture | S72, T931 | |  |  |
| Tibia & ankle fracture | S82 | |  |  |
| Humerus fracture | S422, S423, S424, S427 | |  |  |
| Other fractures | S022– S026, S028, S12, S220– S223, S228, S229, S32, S420–S421, S428–S429, S497, S52, S597, S620–S628, S697, S820, S92, T02, T08, T10, T12, T142, T911, T912, T921, T922, T932 | |  |  |
| Drowning/submersion injuries | T751 | |  |  |
| Dislocations | S030–S033, S131–S133, S231–S232, S331–S333, S430–S433, S530, S531, S630–S632, S730, S830, S831, S930, S931, S933, T03, T092, T112, T132, T143 | |  |  |
| Soft tissue injuries | S034–S035, S134–S136, S16, S230, S233–S235, S290, S335–S337, S390, S434–S437, S46, S532–S534, S56, S633–S637, S66, S731, S76, S832–S837, S86, S932, S934–S936, S96, T064, T095, T115, T135, T146 | |  |  |
| Burns | T20–T32, T95 | |  |  |
| **Cause list (continued)** |  | | |  |
| **Cause name** | **ICD-10 codes** | | |  |
| Medical-related injuries (consequences) | T80–T88, T983 | | |  |
| Residual - injuries | S00, S01, S04, S05, S08–S11, S130, S142–S146, S15, S19, S20, S21, S242–S246, S298, S299, S30, S31, S330, S334, S342–S346, S348, S382, S383, S398, S399, S40, S41, S44, S45, S48, S498, S499, S50, S51, S54, S55, S58, S598, S599, S60, S61, S64, S65, S68, S698, S699, S70, S71, S74, S75, S78, S799, S80, S81, S84, S85, S88–S91, S94, S95, S98, S99, T00, T01, T05, T062, T063, T068, T07, T090, T091, T094, T096, T098, T099, T110, T111, T113, T114, T116, T118, T119, T130, T131, T133, T134, T136, T138, T139, T140, T141, T144, T145, T148, T149, T15–T19, T33–T35, T66–T75, T900, T901, T904, T908, T909, T910, T918, T919, T920, T924, T928, T929, T930,T933, T934, T936, T938, T939, T980, T981, T982 T980–T982 |  |  |  |
| **External causes** | V00–Y98 |  |  |  |
| RTI - motorcyclists | V203 –V209, V213–V219, V223–V229, V233–V239, V243–V249, V253–V259, V263–V269, V273–V279, V283–V289, V294–V299 |  |  |  |
| RTI - motor vehicle occupants | V304–V309, V314–V319, V324–V329, V334–V339, V344–V349, V354–V359, V364–V369, V374–V379, V384–V389, V394–V399, V404–V409, V414–V419, V424–V429, V434–V439, V444–V449, V454–V459, V464–V469, V474–V479, V484–V489, V494–V499, V504–V509, V514–V519, V524–V529, V534–V539, V544–V549, V554–V559, V564–V569, V574–V579, V584–V589, V594–V599, V604–V609, V614–V619, V624–V629, V634–V639, V644–V649, V654–V659, V664–V669, V674–V679, V684–V689, V694–V699, V704–V709, V714–V719, V724–V729, V734–V739, V744–V749, V754–V759, V764–V769, V774–V779, V784–V789, V794–V799, V870–V879, V892, Y850 |  |  |  |
| RTI - pedal cyclists | V103 –V109, V113–V119, V123–V129, V133–V139, V143–V149, V153–V159, V163–V169, V173–V179, V183–V189, V194–V199 |  |  |  |
| RTI - pedestrians | V011, V019, V021, V029, V031, V039, V041, V049, V051, V059, V061, V069, V092, V093, V099 |  |  |  |
| Accidental poisoning - alcohol | X45 |  |  |  |
| Accidental poisoning - drugs | X42–X44 |  |  |  |
| Falls | W00–W19 |  |  |  |
| Drowning | V90, V92, W65–W74 |  |  |  |
| Accidental threats to breathing | W75–W84 |  |  |  |
| Suicide | X60–X84, Y870 |  |  |  |
| Homicide & violence | X85–Y09, Y871 |  |  |  |
| Medical-related injuries (external) | Y40–Y84, Y88 |  |  |  |
| Residual - external causes | V010, V020, V030, V040, V050, V060, V090, V091, V100–V102, V110–V112, V120–V122, V130–V132, V140–V142, V150–V152, V160–V162, V170–V172, V180–V182, V190–V193, V200–V202, V210–V212, V220–V222, V230–V232, V240– V242, V250–V252, V260–V262, V270–V272, V280–V282, V290–V293, V300–V303, V310–V313, V320–V323, V330–V333, V340–V343, V350–V353, V360–V363, V370–V373, V380–V383, V390–V393, V400–V403, V410–V413, V420–V423, V430–V433, V440–V443, V450–V453, V460–V463, V470–V473, V480–V483, V490–V493, V500–V503, V510–V513, V520–V523, V530–V533, V540–V543, V550–V553, V560–V563, V570–V573, V580–V583, V590–V593, V600–V603, V610–V613, V620–V623, V630–V633, V640–V643, V650–V653, V660–V663, V670–V673, V680–V683, V690–V693, V700–V703, V710–V713, V720–V723, V730–V733, V740–V743, V750–V753, V760–V763, V770–V773, V780–V783, V790–V793, V80–V86, V88, V890, V891, V893, V899 , V91, V93–V99, W20–W64, W85–W99, X00–X39, X40–X41, X46–X49, X50–X59, Y10–Y34, Y35–Y36, Y859, Y86, Y872, Y89, Y90–Y98 | |  |  |

For description of **International Classification of Diseases 10^th^ revision** (ICD-10) codes, see World Health Organization (2016).

Four character ICD-10 codes show here without the decimal point (e.g. B942 is B94.2)

CNS Central nervous system

ALRI Acute lower respiratory infection

COPD Chronic obstructive pulmonary disease

RTI Road Transport Injury

SIDS Sudden infant death syndrome

# **Supplementary Table S3. Number of deaths, crude years of life lost (YLL) rates and age-standardised YLL rates by underlying cause of death (UC) and multiple cause weighting (WT) - broad cause groups by sex, Australia, 2015-17**

|  | Males | | | | | Females | | | | |
| --- | --- | --- | --- | --- | --- | --- | --- | --- | --- | --- |
| Broad cause groups | Deaths_UC_ | Crude rate YLL_UC_ | Age standardised rate YLL_UC_ | Crude rate YLL_WT_ | Age standardised YLL_WT_ | Deaths_UC_ | Crude rate YLL_UC_ | Age standardised rate YLL_UC_ | Crude rate YLL_WT_ | Age standardised YLL_WT_ |
| Infectious diseases | 4234 | 1.99 | 2.04 | 1.98 | 2.02 | 4088 | 1.43 | 1.31 | 1.27 | 1.18 |
| Neoplasms | 78461 | 36.83 | 36.90 | 32.57 | 32.63 | 60855 | 28.51 | 26.65 | 25.69 | 24.06 |
| Blood diseases | 736 | 0.35 | 0.36 | 0.49 | 0.50 | 817 | 0.32 | 0.29 | 0.44 | 0.40 |
| Endocrine disorders | 10429 | 4.62 | 4.70 | 6.26 | 6.35 | 9847 | 3.33 | 3.02 | 4.48 | 4.06 |
| Mental & Behavioural disorders | 3461 | 2.42 | 2.45 | 7.18 | 7.25 | 1745 | 1.05 | 1.02 | 2.93 | 2.88 |
| Nervous system diseases | 22140 | 7.12 | 7.50 | 7.46 | 7.85 | 31589 | 7.42 | 6.34 | 7.65 | 6.55 |
| Hearing & vision diseases | 25 | 0.01 | 0.01 | 0.06 | 0.06 | 29 | 0.01 | 0.01 | 0.06 | 0.05 |
| Cardiovascular diseases | 63472 | 23.72 | 24.58 | 24.04 | 24.89 | 65639 | 15.94 | 13.68 | 16.07 | 13.83 |
| Respiratory diseases | 22840 | 7.87 | 8.12 | 7.96 | 8.19 | 22347 | 6.54 | 5.76 | 6.39 | 5.65 |
| Digestive diseases | 6843 | 2.88 | 2.95 | 3.00 | 3.06 | 7902 | 2.41 | 2.15 | 2.31 | 2.08 |
| Skin diseases | 676 | 0.22 | 0.23 | 0.25 | 0.26 | 942 | 0.24 | 0.20 | 0.26 | 0.23 |
| Musculoskeletal conditions | 1422 | 0.52 | 0.54 | 0.86 | 0.89 | 2673 | 0.81 | 0.72 | 1.31 | 1.14 |
| Genitourinary diseases | 4902 | 1.42 | 1.51 | 2.48 | 2.62 | 5813 | 1.35 | 1.14 | 2.07 | 1.77 |
| Maternal conditions | - | - | - | - | - | 25 | 0.04 | 0.04 | 0.05 | 0.05 |
| Perinatal conditions (incl SIDS) | 996 | 2.37 | 2.31 | 2.38 | 2.31 | 779 | 1.82 | 1.90 | 1.84 | 1.92 |
| Congenital conditions | 979 | 1.50 | 1.48 | 1.50 | 1.48 | 835 | 1.23 | 1.26 | 1.21 | 1.24 |
| Injuries - consequences | - | - | - | 0.43 | 0.44 | - | - | - | 0.25 | 0.22 |
| External causes | 20423 | 19.59 | 19.85 | 14.56 | 14.74 | 11694 | 7.49 | 7.38 | 5.67 | 5.56 |

YLL rates are YLL per 1000. There were 4009 deaths in males and 4,787 deaths in females with an ill-defined underlying cause of death; those records have been excluded.

# **Supplementary Table S4. Number of deaths, crude years of life lost (YLL) rates and age-standardised YLL rates by underlying cause of death (UC) and multiple cause weighting (WT) – causes of death by sex, Australia, 2015-17**

|  |  | Males | | | | | | | Females | | | | | | |
| --- | --- | --- | --- | --- | --- | --- | --- | --- | --- | --- | --- | --- | --- | --- | --- |
|  |  | Deaths_UC_ | YLL_UC_ | | | YLL_WT_ | | | Deaths_UC_ | YLL_UC_ | | | YLL_WT_ | | |
|  | Causes |  | Rank | Crude Rate | Age Std Rate | Rank | Crude Rate | Age Std Rate |  | Rank | Crude Rate | Age Std Rate | Rank | Crude Rate | Age Std Rate |
| 1 | Intestinal infections | 339 | 83 | 0.12 | 0.13 | 94 | 0.11 | 0.11 | 550 | 77 | 0.15 | 0.13 | 93 | 0.12 | 0.11 |
| 2 | Tuberculosis | 72 | 108 | 0.03 | 0.03 | 114 | 0.04 | 0.04 | 76 | 109 | 0.03 | 0.03 | 116 | 0.03 | 0.03 |
| 3 | Septicaemia | 2375 | 36 | 0.87 | 0.91 | 46 | 0.68 | 0.70 | 2514 | 30 | 0.74 | 0.66 | 40 | 0.57 | 0.51 |
| 4 | Viral hepatitis | 527 | 55 | 0.43 | 0.44 | 51 | 0.60 | 0.61 | 225 | 74 | 0.16 | 0.16 | 77 | 0.21 | 0.21 |
| 5 | HIV disease | 147 | 79 | 0.13 | 0.13 | 83 | 0.15 | 0.15 | 12 | 114 | 0.01 | 0.01 | 120 | 0.01 | 0.01 |
| 6 | Residual-infectious | 774 | 57 | 0.40 | 0.41 | 62 | 0.40 | 0.40 | 711 | 52 | 0.33 | 0.32 | 58 | 0.32 | 0.30 |
| 7 | Oral cancers | 1839 | 30 | 1.06 | 1.05 | 35 | 0.94 | 0.93 | 742 | 49 | 0.35 | 0.33 | 60 | 0.31 | 0.29 |
| 8 | Oesophagus cancer | 2862 | 21 | 1.52 | 1.50 | 26 | 1.32 | 1.31 | 1070 | 44 | 0.44 | 0.40 | 52 | 0.38 | 0.34 |
| 9 | Stomach cancer | 2138 | 29 | 1.09 | 1.09 | 34 | 0.96 | 0.96 | 1262 | 37 | 0.60 | 0.57 | 43 | 0.54 | 0.51 |
| 10 | Colorectal cancer | 8782 | 5 | 4.20 | 4.22 | 6 | 3.75 | 3.76 | 7557 | 7 | 3.16 | 2.93 | 7 | 2.85 | 2.65 |
| 11 | Liver cancer | 3695 | 16 | 2.07 | 2.05 | 18 | 1.76 | 1.74 | 1905 | 23 | 0.90 | 0.85 | 27 | 0.79 | 0.74 |
| 12 | Gallbladder cancer | 337 | 77 | 0.14 | 0.14 | 91 | 0.12 | 0.12 | 505 | 69 | 0.20 | 0.18 | 83 | 0.17 | 0.15 |
| 13 | Pancreatic cancer | 4501 | 14 | 2.25 | 2.24 | 15 | 1.95 | 1.94 | 4170 | 11 | 1.78 | 1.64 | 12 | 1.56 | 1.44 |
| 14 | Larynx cancer | 537 | 67 | 0.26 | 0.26 | 76 | 0.23 | 0.23 | 73 | 102 | 0.04 | 0.04 | 116 | 0.03 | 0.03 |
| 15 | Lung cancer | 14926 | 3 | 7.14 | 7.07 | 2 | 5.94 | 5.88 | 10216 | 2 | 4.96 | 4.61 | 4 | 4.21 | 3.92 |
| 16 | Malignant melanoma-skin | 2743 | 24 | 1.38 | 1.39 | 27 | 1.26 | 1.27 | 1435 | 31 | 0.73 | 0.69 | 33 | 0.68 | 0.65 |
| 17 | Non-melanoma-skin | 1333 | 51 | 0.53 | 0.54 | 60 | 0.47 | 0.48 | 624 | 73 | 0.17 | 0.15 | 85 | 0.16 | 0.14 |
| 18 | Mesothelioma | 1669 | 41 | 0.68 | 0.67 | 54 | 0.59 | 0.59 | 376 | 74 | 0.16 | 0.15 | 87 | 0.15 | 0.13 |
| 19 | Breast cancer | 86 | 103 | 0.04 | 0.04 | 114 | 0.04 | 0.04 | 8815 | 3 | 4.89 | 4.67 | 3 | 4.62 | 4.41 |
| 20 | Cervical cancer | - | - | - | - | - | - | - | 719 | 40 | 0.53 | 0.53 | 46 | 0.49 | 0.48 |
| 21 | Uterus cancer | - | - | - | - | - | - | - | 1531 | 31 | 0.73 | 0.68 | 37 | 0.65 | 0.61 |
| 22 | Ovarian cancer | - | - | - | - | - | - | - | 2858 | 13 | 1.45 | 1.36 | 15 | 1.31 | 1.23 |
| 23 | Prostate cancer | 9722 | 9 | 3.12 | 3.21 | 9 | 2.91 | 3.00 | - | - | - | - | - | - | - |
| 24 | Kidney cancer | 1857 | 33 | 0.93 | 0.93 | 39 | 0.84 | 0.84 | 999 | 46 | 0.42 | 0.39 | 54 | 0.37 | 0.34 |
| 25 | Bladder cancer | 2268 | 38 | 0.77 | 0.80 | 45 | 0.70 | 0.72 | 897 | 59 | 0.29 | 0.26 | 67 | 0.25 | 0.23 |

**Supplementary Table S4 contd.**

|  |  | Males | | | | | | | Females | | | | | | |
| --- | --- | --- | --- | --- | --- | --- | --- | --- | --- | --- | --- | --- | --- | --- | --- |
|  |  | Deaths_UC_ | YLL_UC_ | | | YLL_WT_ | | | Deaths_UC_ | YLL_UC_ | | | YLL_WT_ | | |
|  | Causes |  | Rank | Crude Rate | Age Std Rate | Rank | Crude Rate | Age Std Rate |  | Rank | Crude Rate | Age Std Rate | Rank | Crude Rate | Age Std Rate |
| 26 | Brain cancer | 2582 | 20 | 1.89 | 1.88 | 19 | 1.73 | 1.72 | 1663 | 16 | 1.16 | 1.13 | 20 | 1.08 | 1.05 |
| 27 | Thyroid cancer | 208 | 89 | 0.11 | 0.11 | 99 | 0.10 | 0.10 | 221 | 88 | 0.10 | 0.09 | 97 | 0.09 | 0.09 |
| 28 | Cancer unknown primary | 4319 | 19 | 1.93 | 1.95 | 22 | 1.64 | 1.66 | 3808 | 14 | 1.42 | 1.29 | 16 | 1.21 | 1.11 |
| 29 | Hodgkin lymphoma | 145 | 92 | 0.09 | 0.09 | 102 | 0.09 | 0.09 | 92 | 98 | 0.05 | 0.05 | 108 | 0.05 | 0.05 |
| 30 | Non-Hodgkin lymphomas | 2634 | 26 | 1.20 | 1.20 | 33 | 1.06 | 1.06 | 1892 | 28 | 0.75 | 0.69 | 33 | 0.68 | 0.62 |
| 31 | Other blood cancers | 6171 | 11 | 2.66 | 2.68 | 11 | 2.38 | 2.40 | 4378 | 12 | 1.76 | 1.62 | 11 | 1.57 | 1.44 |
| 32 | Cancer secondary site | - | - | - | - | 81 | 0.16 | 0.16 | - | - | - | - | 91 | 0.13 | 0.12 |
| 33 | Residual-benign/in situ/uncertain neoplasms | 743 | 58 | 0.38 | 0.38 | 65 | 0.37 | 0.37 | 834 | 52 | 0.33 | 0.31 | 58 | 0.32 | 0.30 |
| 34 | Residual-malignant neoplasms | 2364 | 23 | 1.39 | 1.39 | 28 | 1.25 | 1.25 | 2213 | 17 | 1.14 | 1.08 | 21 | 1.02 | 0.98 |
| 35 | Anaemias | 340 | 79 | 0.13 | 0.13 | 77 | 0.22 | 0.23 | 447 | 86 | 0.12 | 0.10 | 77 | 0.21 | 0.18 |
| 36 | Residual-blood diseases | 396 | 69 | 0.23 | 0.23 | 72 | 0.27 | 0.27 | 370 | 69 | 0.20 | 0.19 | 69 | 0.23 | 0.22 |
| 37 | Disorders of thyroid gland | 81 | 108 | 0.03 | 0.03 | 102 | 0.09 | 0.09 | 305 | 95 | 0.07 | 0.06 | 69 | 0.23 | 0.20 |
| 38 | Diabetes | 7651 | 8 | 3.15 | 3.21 | 5 | 4.11 | 4.18 | 6659 | 9 | 2.14 | 1.92 | 8 | 2.70 | 2.42 |
| 39 | Malnutrition | 148 | 100 | 0.06 | 0.06 | 94 | 0.11 | 0.11 | 166 | 98 | 0.05 | 0.04 | 97 | 0.09 | 0.09 |
| 40 | Obesity | 550 | 54 | 0.46 | 0.46 | 43 | 0.72 | 0.72 | 513 | 48 | 0.36 | 0.36 | 41 | 0.56 | 0.54 |
| 41 | Amyloidosis | 316 | 83 | 0.12 | 0.12 | 94 | 0.11 | 0.11 | 195 | 91 | 0.08 | 0.07 | 102 | 0.07 | 0.06 |
| 42 | Dehydration disorders | 369 | 83 | 0.12 | 0.13 | 83 | 0.15 | 0.15 | 559 | 85 | 0.13 | 0.11 | 89 | 0.14 | 0.12 |
| 43 | Metabolic disorders | 1221 | 45 | 0.64 | 0.65 | 38 | 0.88 | 0.88 | 1282 | 44 | 0.44 | 0.41 | 39 | 0.59 | 0.54 |
| 44 | Residual-endocrine | 93 | 101 | 0.05 | 0.05 | 102 | 0.09 | 0.10 | 168 | 97 | 0.06 | 0.06 | 97 | 0.09 | 0.09 |
| 45 | Alcohol induced diseases | 2796 | 15 | 2.15 | 2.16 | 10 | 2.87 | 2.88 | 945 | 27 | 0.79 | 0.78 | 23 | 0.93 | 0.93 |
| 46 | Substance use disorders | 83 | 92 | 0.09 | 0.09 | 21 | 1.65 | 1.66 | 40 | 102 | 0.04 | 0.04 | 44 | 0.51 | 0.50 |
| 47 | Schizophrenia | 67 | 103 | 0.04 | 0.04 | 61 | 0.42 | 0.43 | 91 | 109 | 0.03 | 0.03 | 81 | 0.18 | 0.17 |
| 48 | Mood disorders | 77 | 111 | 0.02 | 0.03 | 25 | 1.42 | 1.44 | 166 | 102 | 0.04 | 0.03 | 29 | 0.75 | 0.73 |

**Supplementary Table S4 contd.**

|  |  | Males | | | | | | | Females | | | | | | |
| --- | --- | --- | --- | --- | --- | --- | --- | --- | --- | --- | --- | --- | --- | --- | --- |
|  |  | Deaths_UC_ | YLL_UC_ | | | YLL_WT_ | | | Deaths_UC_ | YLL_UC_ | | | YLL_WT_ | | |
|  | Causes |  | Rank | Crude Rate | Age Std Rate | Rank | Crude Rate | Age Std Rate |  | Rank | Crude Rate | Age Std Rate | Rank | Crude Rate | Age Std Rate |
| 49 | Residual-mental/behavioural | 438 | 83 | 0.12 | 0.13 | 40 | 0.82 | 0.84 | 503 | 77 | 0.15 | 0.14 | 41 | 0.56 | 0.55 |
| 50 | Inflammatory diseases - CNS | 129 | 92 | 0.09 | 0.09 | 106 | 0.08 | 0.08 | 126 | 91 | 0.08 | 0.08 | 100 | 0.08 | 0.08 |
| 51 | Systemic atrophies - CNS | 1331 | 37 | 0.78 | 0.77 | 43 | 0.72 | 0.72 | 1145 | 35 | 0.64 | 0.61 | 38 | 0.60 | 0.56 |
| 52 | Parkinson disease | 3314 | 32 | 0.94 | 0.99 | 35 | 0.94 | 0.99 | 1993 | 42 | 0.49 | 0.42 | 46 | 0.49 | 0.42 |
| 53 | Dementia & Alzheimer's disease | 14711 | 7 | 3.44 | 3.77 | 8 | 3.56 | 3.90 | 26142 | 4 | 4.79 | 3.85 | 2 | 4.90 | 3.95 |
| 54 | Multiple sclerosis | 180 | 89 | 0.11 | 0.11 | 94 | 0.11 | 0.11 | 356 | 66 | 0.22 | 0.21 | 74 | 0.22 | 0.21 |
| 55 | Epilepsy | 516 | 52 | 0.49 | 0.49 | 55 | 0.57 | 0.57 | 363 | 55 | 0.32 | 0.32 | 52 | 0.38 | 0.38 |
| 56 | Cerebral palsy | 183 | 70 | 0.22 | 0.22 | 77 | 0.22 | 0.22 | 138 | 74 | 0.16 | 0.17 | 85 | 0.16 | 0.16 |
| 57 | Residual-nervous system | 1776 | 31 | 1.05 | 1.05 | 28 | 1.25 | 1.25 | 1326 | 33 | 0.72 | 0.68 | 25 | 0.82 | 0.78 |
| 58 | Hearing & vision diseases | 25 | 113 | 0.01 | 0.01 | 110 | 0.06 | 0.06 | 29 | 114 | 0.01 | 0.01 | 103 | 0.06 | 0.05 |
| 59 | Chronic rheumatic heart diseases | 431 | 74 | 0.16 | 0.17 | 83 | 0.15 | 0.15 | 726 | 63 | 0.25 | 0.23 | 74 | 0.22 | 0.20 |
| 60 | Hypertension | 585 | 73 | 0.18 | 0.19 | 23 | 1.50 | 1.55 | 1150 | 66 | 0.22 | 0.18 | 14 | 1.36 | 1.16 |
| 61 | Hypertensive diseases | 1982 | 42 | 0.67 | 0.71 | 58 | 0.53 | 0.55 | 3144 | 35 | 0.64 | 0.52 | 48 | 0.44 | 0.36 |
| 62 | Ischaemic heart disease | 32634 | 1 | 12.87 | 13.26 | 1 | 11.40 | 11.74 | 25051 | 1 | 5.95 | 5.07 | 1 | 5.07 | 4.33 |
| 63 | Pulmonary heart diseases | 864 | 56 | 0.42 | 0.42 | 49 | 0.62 | 0.62 | 1261 | 41 | 0.51 | 0.47 | 35 | 0.66 | 0.61 |
| 64 | Non-rheumatic valve disorders | 1994 | 46 | 0.56 | 0.60 | 57 | 0.55 | 0.59 | 2179 | 43 | 0.45 | 0.37 | 51 | 0.42 | 0.35 |
| 65 | Atrial fibrillation | 2333 | 46 | 0.56 | 0.61 | 31 | 1.14 | 1.22 | 4080 | 28 | 0.75 | 0.60 | 18 | 1.12 | 0.92 |
| 66 | Heart failure (specified) | 2380 | 46 | 0.56 | 0.62 | 41 | 0.78 | 0.85 | 3189 | 38 | 0.57 | 0.45 | 27 | 0.79 | 0.64 |
| 67 | Other heart diseases | 3844 | 16 | 2.07 | 2.10 | 13 | 2.07 | 2.11 | 2962 | 17 | 1.14 | 1.06 | 19 | 1.11 | 1.03 |
| 68 | Cerebrovascular disease | 12910 | 4 | 4.21 | 4.41 | 7 | 3.65 | 3.82 | 18611 | 5 | 4.46 | 3.83 | 5 | 3.76 | 3.24 |
| 69 | Artery diseases | 2992 | 28 | 1.13 | 1.16 | 30 | 1.22 | 1.25 | 2629 | 34 | 0.71 | 0.62 | 30 | 0.72 | 0.62 |
| 70 | Phlebitis & thrombophlebitis | 285 | 72 | 0.20 | 0.20 | 74 | 0.24 | 0.24 | 370 | 69 | 0.20 | 0.19 | 69 | 0.23 | 0.22 |
| 71 | Transient cerebral ischaemic attacks | 50 | 113 | 0.01 | 0.01 | 112 | 0.05 | 0.06 | 106 | 112 | 0.02 | 0.01 | 103 | 0.06 | 0.04 |

**Supplementary Table S4 contd.**

|  |  | Males | | | | | | | Females | | | | | | |
| --- | --- | --- | --- | --- | --- | --- | --- | --- | --- | --- | --- | --- | --- | --- | --- |
|  |  | Deaths_UC_ | YLL_UC_ | | | YLL_WT_ | | | Deaths_UC_ | YLL_UC_ | | | YLL_WT_ | | |
|  | Causes |  | Rank | Crude Rate | Age Std Rate | Rank | Crude Rate | Age Std Rate |  | Rank | Crude Rate | Age Std Rate | Rank | Crude Rate | Age Std Rate |
| 72 | Residual-cardiovascular | 188 | 83 | 0.12 | 0.12 | 83 | 0.15 | 0.15 | 181 | 91 | 0.08 | 0.07 | 95 | 0.11 | 0.11 |
| 73 | Influenza | 831 | 65 | 0.29 | 0.30 | 79 | 0.21 | 0.22 | 1188 | 57 | 0.30 | 0.26 | 77 | 0.21 | 0.18 |
| 74 | Pneumonia | 3871 | 27 | 1.19 | 1.27 | 32 | 1.10 | 1.17 | 4795 | 19 | 1.07 | 0.91 | 22 | 0.98 | 0.84 |
| 75 | Other acute lower respiratory infections | 519 | 77 | 0.14 | 0.15 | 81 | 0.16 | 0.17 | 756 | 77 | 0.15 | 0.12 | 87 | 0.15 | 0.13 |
| 76 | COPD | 11764 | 6 | 4.13 | 4.22 | 4 | 4.31 | 4.38 | 10174 | 6 | 3.37 | 3.00 | 6 | 3.31 | 2.95 |
| 77 | Asthma | 430 | 66 | 0.28 | 0.29 | 65 | 0.37 | 0.38 | 894 | 49 | 0.35 | 0.33 | 48 | 0.44 | 0.41 |
| 78 | Bronchiectasis | 362 | 79 | 0.13 | 0.14 | 91 | 0.12 | 0.13 | 814 | 64 | 0.24 | 0.21 | 77 | 0.21 | 0.19 |
| 79 | Pneumonitis | 1244 | 63 | 0.30 | 0.33 | 68 | 0.34 | 0.37 | 1080 | 66 | 0.22 | 0.18 | 69 | 0.23 | 0.19 |
| 80 | Other interstitial respiratory diseases | 2480 | 35 | 0.88 | 0.90 | 42 | 0.76 | 0.78 | 1701 | 39 | 0.55 | 0.49 | 45 | 0.50 | 0.45 |
| 81 | Other diseases of pleura | 155 | 103 | 0.04 | 0.04 | 108 | 0.07 | 0.08 | 110 | 109 | 0.03 | 0.03 | 103 | 0.06 | 0.06 |
| 82 | Residual-respiratory | 1184 | 53 | 0.47 | 0.49 | 59 | 0.50 | 0.51 | 835 | 62 | 0.27 | 0.24 | 63 | 0.29 | 0.26 |
| 83 | Diseases - oesophagus/stomach/duodenum | 851 | 60 | 0.34 | 0.35 | 63 | 0.39 | 0.40 | 884 | 65 | 0.23 | 0.20 | 64 | 0.28 | 0.25 |
| 84 | Other intestinal diseases | 2041 | 40 | 0.70 | 0.73 | 51 | 0.60 | 0.62 | 3175 | 24 | 0.86 | 0.75 | 30 | 0.72 | 0.63 |
| 85 | Diseases of peritoneum | 102 | 101 | 0.05 | 0.05 | 112 | 0.05 | 0.05 | 123 | 98 | 0.05 | 0.04 | 112 | 0.04 | 0.04 |
| 86 | Cirrhosis of the liver | 890 | 49 | 0.55 | 0.55 | 50 | 0.61 | 0.60 | 511 | 59 | 0.29 | 0.28 | 60 | 0.31 | 0.30 |
| 87 | Other diseases of liver | 929 | 49 | 0.55 | 0.55 | 48 | 0.65 | 0.66 | 635 | 52 | 0.33 | 0.31 | 55 | 0.35 | 0.34 |
| 88 | Disorders - gallbladder/biliary tract/pancreas | 908 | 61 | 0.33 | 0.35 | 69 | 0.33 | 0.34 | 1068 | 57 | 0.30 | 0.27 | 66 | 0.26 | 0.23 |
| 89 | Residual-digestive | 1122 | 59 | 0.36 | 0.38 | 63 | 0.39 | 0.40 | 1506 | 49 | 0.35 | 0.30 | 55 | 0.35 | 0.30 |
| 90 | Infections - skin | 457 | 75 | 0.15 | 0.16 | 88 | 0.14 | 0.14 | 592 | 77 | 0.15 | 0.13 | 89 | 0.14 | 0.12 |
| 91 | Residual-skin diseases | 219 | 96 | 0.07 | 0.07 | 94 | 0.11 | 0.12 | 350 | 89 | 0.09 | 0.07 | 91 | 0.13 | 0.11 |
| 92 | Infectious arthropathies | 149 | 103 | 0.04 | 0.05 | 114 | 0.04 | 0.04 | 155 | 102 | 0.04 | 0.04 | 116 | 0.03 | 0.03 |
| 93 | Rheumatoid arthritis | 184 | 96 | 0.07 | 0.07 | 91 | 0.12 | 0.12 | 447 | 82 | 0.14 | 0.12 | 74 | 0.22 | 0.19 |

**Supplementary Table S4 contd.**

|  |  | Males | | | | | | | Females | | | | | | |
| --- | --- | --- | --- | --- | --- | --- | --- | --- | --- | --- | --- | --- | --- | --- | --- |
|  |  | Deaths_UC_ | YLL_UC_ | | | YLL_WT_ | | | Deaths_UC_ | YLL_UC_ | | | YLL_WT_ | | |
|  | Causes |  | Rank | Crude Rate | Age Std Rate | Rank | Crude Rate | Age Std Rate |  | Rank | Crude Rate | Age Std Rate | Rank | Crude Rate | Age Std Rate |
| 94 | Osteoarthritis | 104 | 111 | 0.02 | 0.03 | 99 | 0.10 | 0.11 | 289 | 102 | 0.04 | 0.03 | 81 | 0.18 | 0.14 |
| 95 | Systemic connective tissue disorders | 233 | 79 | 0.13 | 0.13 | 90 | 0.13 | 0.13 | 593 | 59 | 0.29 | 0.27 | 60 | 0.31 | 0.28 |
| 96 | Osteopathies | 378 | 89 | 0.11 | 0.11 | 80 | 0.17 | 0.18 | 786 | 77 | 0.15 | 0.12 | 57 | 0.33 | 0.27 |
| 97 | Residual-musculoskeletal | 374 | 75 | 0.15 | 0.15 | 71 | 0.30 | 0.31 | 403 | 82 | 0.14 | 0.13 | 67 | 0.25 | 0.22 |
| 98 | Glomerular diseases | 173 | 96 | 0.07 | 0.07 | 99 | 0.10 | 0.11 | 112 | 102 | 0.04 | 0.03 | 103 | 0.06 | 0.05 |
| 99 | Renal tubulo-interstitial diseases | 161 | 96 | 0.07 | 0.08 | 108 | 0.07 | 0.07 | 220 | 91 | 0.08 | 0.07 | 100 | 0.08 | 0.07 |
| 100 | Renal failure | 3193 | 34 | 0.90 | 0.97 | 16 | 1.85 | 1.96 | 3515 | 26 | 0.80 | 0.67 | 13 | 1.47 | 1.26 |
| 101 | Urolithiasis | 72 | 108 | 0.03 | 0.03 | 120 | 0.02 | 0.03 | 56 | 112 | 0.02 | 0.02 | 119 | 0.02 | 0.02 |
| 102 | Hyperplasia of prostate | 190 | 103 | 0.04 | 0.04 | 110 | 0.06 | 0.07 | 1910 | 47 | 0.41 | 0.34 | 48 | 0.44 | 0.37 |
| 103 | Residual-genitourinary | 1113 | 62 | 0.31 | 0.33 | 65 | 0.37 | 0.39 | 25 | 102 | 0.04 | 0.04 | 108 | 0.05 | 0.05 |
| 104 | Perinatal conditions (incl SIDS) | 996 | 13 | 2.37 | 2.31 | 11 | 2.38 | 2.31 | 779 | 10 | 1.82 | 1.90 | 9 | 1.84 | 1.92 |
| 105 | Congenital conditions | 979 | 22 | 1.50 | 1.48 | 23 | 1.50 | 1.48 | 835 | 15 | 1.23 | 1.26 | 16 | 1.21 | 1.24 |
| 106 | Traumatic brain injury | - | - | - | - | 114 | 0.04 | 0.04 | - | - | - | - | 120 | 0.01 | 0.01 |
| 107 | Spinal cord injury | - | - | - | - | 123 | 0.01 | 0.01 | - | - | - | - | 127 | <0.1 | <0.1 |
| 108 | Internal & crush injuries | - | - | - | - | 123 | 0.01 | 0.01 | - | - | - | - | 127 | <0.1 | <0.1 |
| 109 | Poisoning-other substances | - | - | - | - | 102 | 0.09 | 0.09 | - | - | - | - | 112 | 0.04 | 0.04 |
| 110 | Hip fracture | - | - | - | - | 118 | 0.03 | 0.03 | - | - | - | - | 112 | 0.04 | 0.04 |
| 111 | Tibia and ankle fracture | - | - | - | - | 126 | <0.1 | <0.1 | - | - | - | - | 127 | <0.1 | <0.1 |
| 112 | Humerus fracture | - | - | - | - | 126 | <0.1 | <0.1 | - | - | - | - | 120 | 0.01 | <0.1 |
| 113 | Other fractures | - | - | - | - | 118 | 0.03 | 0.04 | - | - | - | - | 112 | 0.04 | 0.03 |
| 114 | Drowning/submersion injuries | - | - | - | - | 126 | <0.1 | <0.1 | - | - | - | - | 127 | <0.1 | <0.1 |
| 115 | Dislocations | - | - | - | - | 126 | <0.1 | <0.1 | - | - | - | - | 127 | <0.1 | <0.1 |
| 116 | Soft tissue injuries | - | - | - | - | 126 | <0.1 | <0.1 | - | - | - | - | 127 | <0.1 | <0.1 |
| 117 | Burns | - | - | - | - | 126 | <0.1 | <0.1 | - | - | - | - | 127 | <0.1 | <0.1 |
| 118 | Medical-related injuries (consequences) | - | - | - | - | 123 | 0.01 | 0.01 | - | - | - | - | 120 | 0.01 | 0.01 |

**Supplementary Table S4 contd.**

|  |  | Males | | | | | | | Females | | | | | | |
| --- | --- | --- | --- | --- | --- | --- | --- | --- | --- | --- | --- | --- | --- | --- | --- |
|  |  | Deaths_UC_ | YLL_UC_ | | | YLL_WT_ | | | Deaths_UC_ | YLL_UC_ | | | YLL_WT_ | | |
|  | Causes |  | Rank | Crude Rate | Age Std Rate | Rank | Crude Rate | Age Std Rate |  | Rank | Crude Rate | Age Std Rate | Rank | Crude Rate | Age Std Rate |
| 119 | Poisoning-alcohol | - | - | - | - | 120 | 0.02 | 0.02 | - | - | - | - | 120 | 0.01 | 0.01 |
| 120 | Poisoning-opioid | - | - | - | - | 120 | 0.02 | 0.02 | - | - | - | - | 120 | 0.01 | 0.01 |
| 121 | Residual-injuries | - | - | - | - | 83 | 0.15 | 0.15 | - | - | - | - | 103 | 0.06 | 0.06 |
| 122 | RTI: motorcyclists | 590 | 39 | 0.76 | 0.76 | 46 | 0.68 | 0.69 | 45 | 98 | 0.05 | 0.05 | 108 | 0.05 | 0.05 |
| 123 | RTI: motor vehicle occupants | 1676 | 18 | 2.04 | 2.05 | 17 | 1.81 | 1.82 | 782 | 24 | 0.86 | 0.88 | 26 | 0.80 | 0.82 |
| 124 | RTI: pedal cyclists | 87 | 95 | 0.08 | 0.08 | 106 | 0.08 | 0.08 | 9 | 114 | 0.01 | 0.01 | 120 | 0.01 | 0.01 |
| 125 | RTI: pedestrians | 291 | 63 | 0.30 | 0.30 | 73 | 0.25 | 0.25 | 159 | 82 | 0.14 | 0.14 | 93 | 0.12 | 0.12 |
| 126 | Accidental poisoning-alcohol | 188 | 71 | 0.21 | 0.21 | 88 | 0.14 | 0.14 | 74 | 95 | 0.07 | 0.07 | 108 | 0.05 | 0.05 |
| 127 | Accidental poisoning-drugs | 2226 | 10 | 2.77 | 2.82 | 20 | 1.70 | 1.73 | 947 | 20 | 1.06 | 1.07 | 35 | 0.66 | 0.67 |
| 128 | Falls | 3847 | 25 | 1.28 | 1.35 | 35 | 0.94 | 1.00 | 4268 | 22 | 0.93 | 0.79 | 32 | 0.69 | 0.58 |
| 129 | Drowning | 518 | 43 | 0.66 | 0.65 | 55 | 0.57 | 0.57 | 137 | 72 | 0.19 | 0.19 | 83 | 0.17 | 0.18 |
| 130 | Accidental threats to breathing | 332 | 67 | 0.26 | 0.26 | 74 | 0.24 | 0.24 | 221 | 86 | 0.12 | 0.12 | 95 | 0.11 | 0.11 |
| 131 | Suicide | 6850 | 2 | 8.04 | 8.11 | 3 | 5.23 | 5.27 | 2282 | 8 | 2.64 | 2.69 | 10 | 1.63 | 1.67 |
| 132 | Homicide & violence | 487 | 43 | 0.66 | 0.66 | 51 | 0.60 | 0.60 | 231 | 56 | 0.31 | 0.31 | 64 | 0.28 | 0.29 |
| 133 | Medical-related injuries (external) | 261 | 83 | 0.12 | 0.12 | 70 | 0.31 | 0.31 | 221 | 89 | 0.09 | 0.08 | 69 | 0.23 | 0.22 |
| 134 | Residual-external causes | 3070 | 12 | 2.43 | 2.47 | 14 | 2.01 | 2.04 | 2318 | 21 | 1.03 | 0.98 | 24 | 0.85 | 0.81 |

YLL rates are YLL per 1000. Lowest rank was assigned in case of tied rates.
